# Development and implementation of work-oriented clinical care to empower patients with kidney disease: an adapted intervention mapping approach

**DOI:** 10.1186/s12913-023-09307-9

**Published:** 2023-04-01

**Authors:** Haitze J. de Vries, Wim S. Sipma, Ron T. Gansevoort, Sandra Brouwer, Annemieke Visser

**Affiliations:** 1 Department of Health Sciences, Community and Occupational Medicine, University of Groningen, University Medical Center Groningen, Hanzeplein 1, RB 9700 Groningen, the Netherlands; 2 Erasmus University Rotterdam, Erasmus School of Health Policy & Management, Rotterdam, the Netherlands; 3 Department of Internal Medicine, University of Groningen, University Medical Center Groningen, Groningen, the Netherlands; 4 Department of Health Sciences, University of Groningen, University Medical Center Groningen, Applied Health Research, Groningen, the Netherlands

**Keywords:** Work ability, Kidney disease, Work-oriented clinical care, Program development, Adapted intervention mapping

## Abstract

**Background:**

Many people with chronic kidney disease (CKD) have problems to stay at work. Patients and health care professionals (HCPs) see the potential benefit of work-oriented clinical care, yet this care is not manifested in current practice. The aim of this study was to develop and implement a program called work-oriented clinical care for kidney patients (WORK) to support sustainable work participation.

**Methods:**

An adapted version of Intervention Mapping (AIM) was used for the systematic development of work-oriented care in a hospital. Based on the needs of patients and (occupational) health professionals, and in close cooperation with both, a theoretical and empirically based program was developed. Feasibility and clinical utility were assessed among patients with CKD, HCPs and hospital managers. To increase the chances of successful implementation we focused on determinants related to the innovation, the users, the organization (hospital), and socio-political context.

**Results:**

We developed, implemented, and pilot-tested WORK, an innovative program consisting of a care pathway in the hospital that targets patients with work-related questions and tailors the support they receive to their needs. Several practical tools were developed and an internal and external referral structure with a focus on work was implemented. A labor expert was deployed to the hospital to support patients and HCPs with simple work-related questions. The feasibility and clinical utility of WORK were rated positively.

**Conclusions:**

This work-oriented clinical care program provides HCPs in the hospital with the necessary tools to support patients with CKD in dealing with work challenges. HCPs can discuss work with patients at an early stage and support them in anticipating work-related challenges. HCPs can also bridge the gap to more specialized help if necessary. WORK has the potential for wider application in other departments and hospitals. So far, the implementation of the WORK program was successful, though structural implementation may be challenging.

**Supplementary Information:**

The online version contains supplementary material available at 10.1186/s12913-023-09307-9.

## Background

Where in the past having a paid job was often regarded as a risk factor for health, it is now regarded as a determinant of health [1–3]. Work gives meaning and structure to people’s lives and leads to better health outcomes [4, 5]. This includes people with kidney disease [6, 7], for whom employment significantly contributes to general well-being, mental health, and quality of life [8, 9]. In this study we focus on working age patients with chronic kidney diseases (CKD) categories G3b-5. The ability of many patients with CKD to maintain their work is severely limited by physical and cognitive complaints [10, 11] and, in case of dialysis, by the necessity for timely and intensive medical treatment. Patients experience uncertainty about the course of the illness, their employers’ responses, and legislation and regulations [9] and have an increased risk of long-term absenteeism, loss of employment, and loss of income [12]. These patients experience work as a continuous learning process with a constant need for adjustments [13, 14].

Despite these challenges, people with diagnosed CKD across different categories indicate that sustainable work participation is an important goal and many are highly motivated to achieve this goal [13, 15]. Therefore, it is important to give more attention to the work-related challenges people with CKD may experience. This importance is increasingly recognized within curative care [16]. HCPs in the hospital may play an important role in preparing patients with CKD early regarding how treatment may interfere with work and signaling other problems that may arise related to work participation [12]. In addition, HCPs may refer to appropriate work-oriented support outside the hospital. However, current guidance and support in terms of work retention for patients with CKD is still inadequate, and many patients have no place to go with their work-related questions [9]. Moreover, HCPs experience a lack of referral options. For example, though nephrology care has the potential to support patients dealing with the challenges of working with a kidney disease (such as advising them about the choice of dialysis modality [17]), this is not established in current practice [16]. In conclusion, so far there is no culture of work-oriented medical care in hospitals [4, 18–20].

The primary aim of this study was to develop and implement work-oriented clinical care for kidney patients (WORK). For that purpose, we aimed to create a culture that recognizes the importance of HCPs providing work-oriented medical care in the hospital, such that attention to the impact of CKD on the work life of patients becomes a more natural part of care. Feasibility and clinical utility were assessed among patients with CKD, HCPs and hospital managers. The secondary aim was to strengthen the self-direction of patients with CKD by engaging and empowering them.

## Methods

### Design

In this study, we used the adapted version of intervention mapping (AIM) [21, 22]. AIM is guided by the six steps of Intervention mapping for development, implementation and evaluation of theory and evidence-based health promotion intervention [23]. In the adapted version, the principles of participatory action research are added, where all stakeholders are working and learning together and have a fair say in the fulfillment of the innovation. Producing and applying knowledge with all stakeholders at the same time provides insight into what may and what may not work. This strategy increases the chance of producing a care innovation that is suitable, acceptable, feasible, and effective and thereby increases the chances of successful adoption and implementation [21]. This study takes a person-centered perspective, putting patients, their way of living, and their personal contexts at the forefront. In addition, this study sees the patient as an active participant in care [24].

### Study setting

Our study was initiated by the University Medical Centre Groningen (UMCG), a leading hospital in the northern part of the Netherlands. The Department of Nephrology offers care for patients with early categories of kidney failure and dialysis and transplantation for patients with kidney failure. The UMCG has a full-service Nephrology Department with close connections to other hospitals – both regionally and nationwide. Furthermore, within the hospital setting a separate dialysis unit offers treatment for patients who need less complicated care. This unit has multiple centers around the city of Groningen.

The Dutch healthcare system provides every citizen with full coverage of medical costs regardless of age, employment status, or health care status. It is a hybrid system with central governmental regulation and private insurance companies that contract private healthcare providers such as hospitals. The social security system for patients who lose their jobs for medical reasons is separate from the health care system. Employers are obliged to pay employees during sick leave, for a maximum of two years. Occupational physicians, who are not involved in the medical treatment of patients, support and advice workers and employers on issues related to work and health, in order to reduce long-term sick leave and work disability and to facilitate sustainable employment. During the first two years of sick leave, a labor expert may be involved, who can advise on reintegration options and empower workers to find the right information or support. After two years of sick leave, the employee can be fired. Employees may then receive financial support from a governmental agency called the Institute for Employee Insurance (UWV). To get admitted to the employee benefits scheme (WIA), an insurance physician from UWV must rule on the employee’s ability to work. In general, if the employee has no work ability, they can be paid out 70% of the last earned wages, up to a maximum of 100%. When people are partially unable to work, the system encourages them to work: if you have a job, you get paid more and the allowance is only partly reduced. However, the system for self-employed workers, approximately 17% of the Dutch workforce [25], is different. Self-employed workers have no access to the WIA. While many choose private insurance to avoid loss of income, a large group (40%) do not [26], partly because these insurance premiums are high. Thus, if workers get sick and not able to work, they risk living without income and, as a consequence, may rely on their savings during their sick leave.

### Establishing project organization and stakeholder participation

The project was organized by establishing a Core team, a Taskforce, and an Advisory Board (see Additional file 1). Agreements were made about decision-making, collaboration, learning, and reflection. Participation of stakeholders was secured at four relevant perspectives: 1) patients with all categories of CKD; 2) HCPs in the hospital (nephrologists, kidney care nurse specialists, social workers); 3) occupational health professionals (occupational health physician, labor expert, insurance physician); and 4) researchers with a focus on work and health. Four project leaders of the Core team, who represented all four stakeholder perspectives, were responsible for the project’s progress. Each individual member of the Core team acted as coordinator and linking pin to the Taskforce, in which several additional representatives of each of the various stakeholder groups participated. An Advisory Board was formed in order to include knowledge from employer representatives, other hospitals, professional groups, and knowledge institutes and to facilitate the possibility of later extrapolating the knowledge acquired during this project to nephrological departments in other hospitals or to care of other chronic diseases. The Advisory Board met twice during the project, at the beginning and at the end. Furthermore, to seek advice and best practices, regular consultations were held with other groups in the Netherlands that are involved in the development of work-oriented clinical care, including the Maastricht University Medical Center (MUMC), the Radboud University Medical Center (RUMC), and the Fit for Work platform, a group committed to job retention for people with chronic conditions.

### Data collection

For the development of the intervention, we carried out the first five prescribed phases of AIM and made a start on the sixth phase [21]. AIM has an iterative nature that allows for moving back and forth between phases and incorporating the feedback of stakeholders, with each phase based on the previous phases. Applying AIM consists of several AIM meetings with the Core team and Taskforce or Advisory Board (see Table 1). In the years 2020–2021, the Taskforce met 10 times. As a result of COVID-19, some meetings were organized digitally or in hybrid form and some of these AIM meetings took place in subgroups. From the onset, we realized that power dynamics could interfere with the group process, where doctors, nurses and patient were supposed to work together as a group. During the first AIM meetings an external facilitator smoothened the process between the group members, who introduced themselves to each other and shared their background and their interests. This was done in an informal setting and helped to set the rules of open communication and equal contribution. Each meeting lasted approximately two hours. The Core team took turns leading sections of each meeting and taking notes, and jointly completed a debriefing form at the conclusion of the session, so that the results could be included in the further development of the innovation. During the meetings all stakeholders jointly discussed themes, shared knowledge, reflected, learned, and worked together to develop the program. In later phases of development, the role of the Taskforce was to assess (intermediate) products, provide input, contribute with ideas, and validate the WORK program.

**Table 1:** Intervention mapping process (adapted from Belansky et al., 2013 [22])

AIM phases	Meeting	Who	Topic
**Phase 1: Formulating program goals**	*1*	Core team and Taskforce	Personal introduction Introduction of the project Making agreements about decision-making, collaboration, learning and reflection
	*2*	Core team and Taskforce (in subgroups)	Needs assessment Agree on a definition of work-oriented medical care Validating and refining scientific knowledge about labor participation Identifying gaps in work-oriented care in the hospital Joint formulation and reporting of program goals
**Phase 2: Defining change objectives**	*3/4*	Core team	Stating expected outcomes for behavior and environment Specifying performance objectives (what or who needs to change)
			Construct matrices and prioritizing of change objectives
**Phase 3: Selecting theory-based methods and practical applications**	5	Core team and Advisory board	Seek advice and best practices with regard to the development and implementation of work-oriented medical care
	*6*	Core team and Taskforce	Generating program themes, components, scope, and sequence Choose theory- and evidence-based change methods Selecting practical applications and best practices to achieve change objectives
**Phase 4: Developing the program**	*7–8*	Core team and Taskforce	Converting knowledge into a concrete action plan (who will do what, when and how)
			Identifying conditions, barriers, and challenges Pilot testing, refining and adjusting materials
	*9*	Core team and Advisory board	Ask for response from the Advisory board on practical products, planned implementation, and seek advice about dissemination
**Phase 5: Adoption and implementation of the program**	*10*	Core team	Design implementation plan and strategies Identifying potential users/implementers
			Defining outcomes of adoption and implementation
**Phase 6: Reflection and evaluation**	*All meetings*	Core team	Reflection on the process Feasibility and clinical utility

In phase six, the first evaluation of WORK focused on feasibility [27] and utility of the program for clinical practice [28]. Feasibility was defined as the extent to which the program proved to be feasible in practice, and clinical utility was defined as the extent to which the program had utility or added value for HCPs and patients, as well as the advantages and disadvantages of working with the WORK program. To explore feasibility and clinical utility, short questionnaires were given to patients with CKD and short semi-structured qualitative interviews were conducted with HCPs and managers in the hospital (Additional files 3 and 4). This study of the effectiveness of the WORK program is not within the scope of the current paper and will be presented when available in a separate paper.

### Data analysis

Minutes and logs of each AIM meeting were kept, and data was transcribed. Subsequently, data was analyzed, and a meeting report provided to the participants. During each following meeting there was reflection in the Core team to validate the findings. The level of implementation (fully, partially, or not implemented) was independently scored by HdV, AV, and WS and discussed until agreement was found. Evaluation of and reflection on the process was conducted during meetings with the Core team. For the evaluation of feasibility and clinical utility, qualitative thematic analyses and descriptive quantitative data analyses were applied.

## Results

The WORK project was conducted from February 2020, with the organization of a first meeting with the Taskforce, to November 2022, when the project ended with the evaluation with patients and HCPs. The results are presented here per phase, with the corresponding AIM meetings that took place.

### Phase 1: Formulating program goals

Phase 1 involved a needs assessment to identify the needs of patients with CKD and HCPs related to work-oriented care in the hospital. The previously conducted CKD@Work study [9, 11] resulted in themes such as the meaning of work [9], barriers and facilitators of sustained employment [9], and associations between patient characteristics, type of treatment and employment status [10, 11]. These themes were discussed, validated and, if necessary, refined or supplemented, taking into account the different needs that emerged from the different perspectives. *Patients with CKD* indicated that work is important for participation and income, but that they often have difficulties to stay at work and dealing with complicated administrative procedures in the event of long-term absenteeism. In this regard, patients currently experience little support from the hospital, and they report that more attention to work is warranted. They indicated that the type of treatment they receive can influence their ability to work (see for examples Table 2) and that doctors often do not take interference of treatment on work into account. Patients therefore emphasized the need for work-oriented clinical care and involvement of the nephrologist. Good communication between nephrologist and occupational physician was also recommended, which is rarely the case in practice. Patients also indicated that it is important to them that their own agency be strengthened. The *HCPs of the hospital* saw it as their task to pay attention to work and to be more aware of the value of work for patients and the role they can play in signaling work challenges, preferably early in the disease process. In particular, nephrologists reported lack of time, knowledge, skills, and referral options, which hinders them from discussing work with patients. They therefore need work-oriented care that can be easily integrated into healthcare, takes little time, and has an easier referral process for patients. There is also a need for easy ways of exchanging information between professionals inside and outside the hospital, a process that is currently encumbered by strict privacy legislation. The *occupational health professionals* believe that the current focus in the hospital is too centered on medical treatment and not centered enough on the possibilities for patients to participate optimally in work. Occupational health professionals expressed the need to broaden the scope of clinical care and to develop work-oriented clinical care intended to facilitate access to the occupational healthcare already available outside the hospital.

**Table 2:** Examples to overcome interference of CKD treatment and work

Health care professionals in the hospital can make an effort to coordinate work and treatment. For example:
-Transplant or dialysis schedules can be planned in consultation with the patient, who may have preferences related to work. Dialysis can be performed at times outside of working hours that are more feasible for people still working (e.g., evenings or weekends)
-Exercise shared decision making about dialysis modality. Sometimes an ArterioVenous Fistula / ArterioVenous Graft (AVF/AVG) is needed, but if the patient is on the waiting list for transplant, consider working with a jugular central venous catheter for longer than normal. Peritoneal or hemodialysis: peritoneal dialysis is more flexible, especially the nocturnal form (APD). Discuss the fact that the peritoneum is a wearable artificial kidney
-AVF/AVG placement is always in the least used arm, but that arm must be spared for the rest of life. This limits patients who still (want to) do physical work, or play sports (e.g., tennis)
-Before and after transplant, (vocational) rehabilitation can be recommended. In general, the fitter patients are on the operating table, the faster they will recover and be able to resume work
In all cases, choices must be logistically feasible and medically justified.

In the second AIM meeting, agreement was reached regarding what work-oriented clinical care entails. Work-oriented medical care was defined as “care in the hospital aimed at supporting sustainable employability of patients with CKD who work or want to work and should focus on patients in different categories of the disease (pre-dialysis, dialysis, and transplantation)”. Based on the first meeting, it was established that the benefit of work-oriented care mainly lies in targeting patients and offering simple support and appropriate referral. This entails attention to adapting CKD treatment to the patients’ work context in cooperation with the nephrologist, providing education and information about working with CKD, and, if needed, referring the patient to specialized work-oriented care inside or outside the hospital. We see the hospital as a bridge between patients and work-related care, preferably in an early stage of the disease. Offering work and mediating to find work are not part of the WORK program.

To realize the WORK program, we refined and established two aims: 1) *Structural embedding of work-oriented care in the hospital*. This goal aimed to create a culture in which the importance of work-oriented medical care is recognized by HCPs ( i.e., nephrologists, nurses and social workers), and where attention to work becomes a more natural part of care in the hospital; 2) *Strengthening self-direction of patients with CKD to deal with work challenges*. The ultimate aim of the program was for patients with CKD to gain better work ability, sustainable employability, and financial stability, which will in turn contribute to better health.

### Phase 2: Defining change objectives

**Fig. 1:**
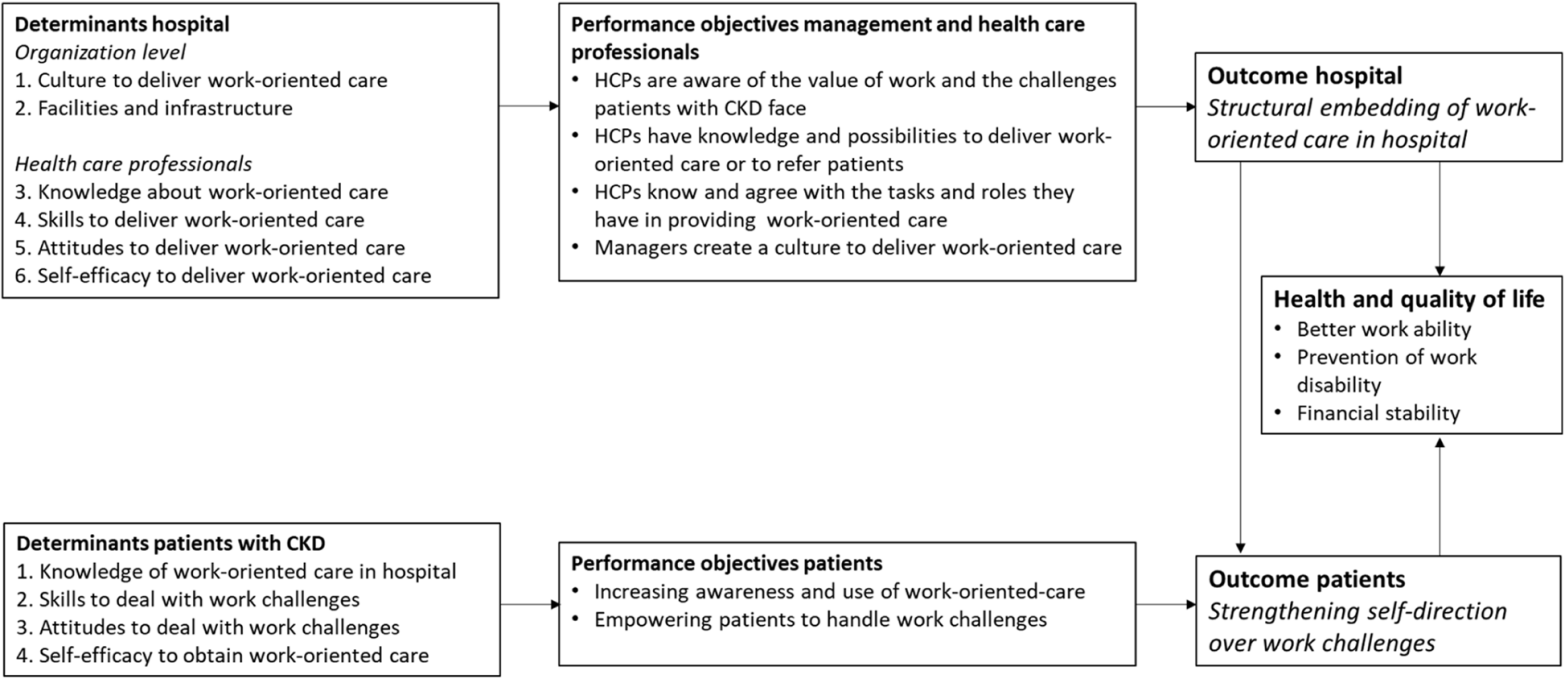
Logic model of change

In phase 2, during the third and fourth AIM meetings, expected outcomes for the hospital (managers and HCPs) and patients with CKD were discussed. In addition, performance objectives were defined, which indicate what needs to be done to accomplish the outcomes. A logic model of change was developed (Fig. 1) to visualize the determinants that need to be considered in developing the program, the anticipated performance objectives, and the potential outcomes for both the hospital and patients with CKD.

Based on the logic model of change, a matrix of change objectives was constructed (Additional file 2). The performance objectives of both the hospital (management, HCPs) and patients with CKD are presented in the first column of the matrix. The associated change objectives in the following columns indicate what hospital management, HCPs and patients with CKD need to learn or change to achieve the performance objectives. To enable HCPs to learn to apply the WORK program and embed it in the hospital, the Attitude-Social influence-Efficacy (ASE) model [29] was selected and supplemented with knowledge, skills, and facilities. We used the ASE-model because it has proven to be useful for predicting and explaining behavior change among HCPs [30]. These ASE-determinants were translated into change objectives for the work-oriented support intervention.

### Phase 3: Selecting theory-based methods and practical applications

To accomplish the change objectives of the hospital (management and HCPs) and patients with CKD, several theory-based methods and practical applications were selected (see Table 3). The selected methods were derived from the literature [23], whereas the practical applications were developed based on consultation with stakeholders during AIM meetings and with the Taskforce and Advisory Board. Some practical applications were also inspired by other initiatives and best practices on work-oriented care according to the Support and Advice Center of the Dutch Association for Kidney Patients, MUMC [31], RUMC [32], and the Fit for Work platform.

**Table 3:** Overview of selected theoretical methods and practical application for use in the program

**A.** Hospital: HCPs and management		
**Change objectives**	**Theory-based methods**	**Practical application**
Knowledge	Information about work-oriented care	Information (leaflets, mail, letters, intranet, infographics) is distributed to inform HCPs about the program Awareness for work-oriented care is raised via digital screens in waiting areas of the hospital Presentations for HCPs and management
	Awareness	Clarity is provided about specific tasks and roles of HCPs in delivering work-oriented careNational reports and guidelines about the need for work-oriented medical care are sharedPersonal stories of patients who struggle with work challenges are shared (personas)
	Sense making	HCPs are involved and ideas around work-oriented care are exchangedPresentations for middle and higher management to create meaning through dialogue
Skills	Competence training	HCPs learn how to deliver work-oriented care through informal training Tools were developed for HCPs (e.g., three work questions, flowchart on work-oriented care, referral cards, folders for employers)
	Case descriptions	Privacy rules for referral to and consultation with work-oriented specialists were made explicit
	Targeting	HCPs learn how to deliver work-oriented care through case descriptionsHCPs learn to identify patients with CKD who are working and need support
Attitude	Modeling	Early adapters are identified and deployed as ambassador
	Public commitment	Ambassadors engage themselves to deliver work-oriented care and announce that decision to colleagues
	Consciousness raising	Personal stories (personas) are distributed of patients who struggle with work challengesLabor expert provides feedback on the results of work-oriented care
Social norms	Goal setting	Work-oriented care is part of the vision/mission of the department/hospital
	Modeling	Managers and supervisors share the need for work-oriented care with HCPsManagers facilitate work-oriented medical careHCPs are reinforced by the achievements of colleagues
	Increasing stakeholder influence	Meeting is organized with a large insurer (MENZIS) that is strongly connected to the hospitalCooperation with the Dutch Association for Kidney Patients
	Nudging	Desk calendars are handed out to remind HCPs to take work into account
Self-efficacy	Structural redesign	Attention for work is integrated in (administrative) systems and protocols Sufficient expertise in the hospital is arranged Work-oriented care is integrated into electronic patients file systems A flow diagram is developed in which targeting, tailoring, and referral with regard to work-oriented care is explained Time needed by nephrologists for work-oriented care has been kept to a minimum
	Feedback	HCPs receive feedback from a labor expert about the results of work-oriented careWork-oriented care is made part of regular meetings in the hospital
	Task clarity	Clarity of tasks and responsibilities in work-oriented care is established
	Mobilizing support	Increase expertise by hiring a labor expert who is present and visible in the department
**B.** CKD patients		
**Change objectives**	**Theory-based methods**	**Practical application**
Knowledge	Information about work-oriented care	Information (leaflets, mail, infographics, informational letters) is distributed to inform patients with CKD about work-oriented care in the hospital Video screens are applied to be used in waiting areas of the hospital Article published in a journal for patients with CKD Comprehensibly formulated knowledge is used
	Awareness/Discussion	Nephrologists discuss the interference of treatment and work with patientsSocial workers or nurse specialists discuss the importance of being proactive with patientsPatients are informed about their responsibilities and rights with regard to social securityHCPs discuss advantages and disadvantages of disclosure of disease at work
Skills	Guided practice and skills enhancement	Empower patients to ask questions about work challenges Support patients in the decision to disclose or not disclose their disease with an employer Support and prepare patients in the consults they have with employer, occupational physician, and labor expert
	Shared decision-making	Patients discuss how to deal with interference of treatment and work with the nephrologistPatients discuss how to prepare for an operation and how to work on recovery
Attitude	Consciousness raising	Discuss the possible consequences of CKD for work with patients Stimulate patients to ask questions about work challenges Motivate patients to make use of work-oriented care in the hospital and ask for support Challenge patients to be pro-active when it comes to work challenges
	Role modeling	Share cases or personas of patients who managed to stay at work with CKD
	Discussion	Discuss patients’ responsibilities in the return-to-work trajectoryDiscuss the consequences of work disability and job loss
Self-efficacy	Modeling	Distribute personal stories (personas) of patients who solved their work challenges
	Practical support	Share contact information for questions (Dutch Association for Kidney Patients)Provide information about the social security systemRefer patients to a labor expert who can be easily consultedRefer patients to the occupational physician or other work-focused specialists outside the hospital
	Empowerment	Provide a folder about CKD to share with the employerHCPs and patients with CKD discuss work challenges and how to overcome them

*HCPs* Health care professionals, *CKD* Chronic kidney disease

### Phase 4: Developing the program

During AIM meetings with the Taskforce, the results of phase 3 were converted into concrete tools or actions.

#### Structural embedding

Most hospital managers and HCPs endorsed the need for work-oriented care. However, a supportive culture, facilities, and infrastructure to provide such care was lacking. Nephrologists mentioned that they lacked time, knowledge, and referral options. Given patients’ wishes to involve nephrologists, as well as the Royal Dutch Medical Association’s (KNMG) recommendation to incorporate work participation as an essential part of medical care, the Core team decided to involve nephrologists. Subsequently, the aforementioned barriers were anticipated on by developing three work questions (takes little time and requires no knowledge) and by involving high-quality work-related expertise of a labor expert from the Center for Rehabilitation UMCG for half-day per week to offer expertise and referral options. The three work questions were integrated in workflow processes and administrative systems of nephrologists, nurses, and social workers in the hospital. As a reminder, the same three work questions were distributed via conversation cards and desk calendars. The basic principle was that the three work questions should be asked to all new patients early in the disease process and then repeated at later moments. In consultation with the HCPs, we decided to give the social worker an important task in coordination (though in other departments this could instead fall to the nurse or nurse specialist). Based on the advice of the relevant work-related experts, we developed an indexation scheme by which social workers can quickly determine when referral to a labor expert is necessary, especially for patients at extra risk of dropout or problems at work. We deployed a labor expert in order to offer this expertise within the hospital. In addition to the labor expert, patients could be referred to the Support and Advice Center of the Dutch Association for Kidney Patients for more long-term support. Referral to a rehabilitation center was also made easier (in case of complex work-related questions).

#### Strengthen self-direction of patients with CKD

To strengthen the self-direction of patients with CKD, we first of all looked at which existing best practices and informational materials were available that fit the change objectives formulated in Phase 3. For example, materials from the Dutch Association for Kidney Patients and the Fit for Work platform were adopted. Additionally, with the help of a graphic designer, visually appealing and well-arranged program materials were designed. Patients were encouraged to think about work and, if desired, to discuss this with their HCP via brochures (“Keep working, how do I do it?”), discussion cards with the three work questions, posters, messages on the video screens at the outpatient clinic, and information on the program that was added to the invitation letters. Patients who indicated a need for information or support based on the three work questions were referred to the social worker who indicated (on the basis of risk factors) and provided tailor-made care. In many cases, this meant a referral to the labor expert who was present at the outpatient clinic for a fixed part of the week. Tools were also developed to give patients more knowledge about what to expect when working with CKD and how to prepare for it, for example to improve knowledge about legislation and regulations. The patients involved indicated that patients should not be overloaded with written materials, so we limited those materials to what is necessary. For legal questions or support, external referral opportunities were created. In addition, for complex cases referral to vocational rehabilitation was facilitated.

### Phase 5: Adoption and implementation of the program

Phase five yielded the development of an implementation plan (i.e., strategies to enable adoption, implementation, and continuation of the program). For this purpose, we used the framework and measurement instrument for determinants of innovations (MIDI) [33].

We anticipated on four categories of determinants that may influence adoption, implementation, and continuation of the program: 1) characteristics of the innovative WORK program, 2) future users (HCPs in the hospital) and end users (patients with CKD), 3) the organization (UMCG and Department of Nephrology), and 4) the socio-political context [33].

The target outcome with regard to adoption is that HCPs understand and endorse the intention of the WORK program, are positive about the rationale and purpose, and are willing to apply it in practice. The ideal implementation outcome is that HCPs involved apply the WORK program in their clinical practice with patients with CKD. The target outcome for continuation is that the WORK program is carried on within the Nephrology Department and expanded to other departments of the hospital. Table 4 shows the authors’ retrospective assessment of the extent to which we were able to ensure the achievement of these determinants on a three-point scale (+ , ± , -).

**Table 4:** Determinants for successful implementation of the WORK program

**1. Innovation** - Compatibility with current care (+) - Complexity (+/-) - Procedural clarity (+) - Appealing (+) - Relevance for patients (+) - Expected advantage for patients (+) - Visibility of outcomes (+/-) - Users involved in development (+) - Prevalence (+/-) **2. Users (HCPs)** - Awareness of content of innovation (+) - Knowledge and skills (+) - Subjective norms (+) - Job perceptions (+/-) - Personal benefits (+/-) - Social support (supervisors, colleagues) (+/-) - Self-efficacy (+) - Overload (-) - Opposing goals or interests (+/-)	**End users (patients with CKD)** - Knowledge about the program (+/-) - Patient cooperation (+) - Patient satisfaction (+) **3. Organization** - Formal ratification by management (+) - Vision, person-centered care (+/-) - Material resources and facilities (+) - Financial resources (+/-) - Time available (+/-) - Use of opinion leaders, ambassadors (+/-) - Use of a coordinator, project group (+/-) - Information accessible about use of innovation (+) - Feedback to users about innovation process (+) - Turbulence in organization, COVID-19 pandemic (-) **4. Socio-political context** - Fit with existing legislation and regulations (+/-) - Work-focused care on political agenda (+/-) - Participatory society, emphasis on self-reliance +) - Cuts to innovation budgets (-)

#### Innovation

The WORK program was practically and technically compatible with current clinical care. Appealing tools and products were developed that fit in the current treatment and care processes and these were acceptable for clinical practice. For example, we limited the nephrologists’ time investment in this program to a minimum. This is important because adoption in clinical practice is a prerequisite for further implementation. The WORK program is clearly described in a flowchart and is not complicated to understand. After getting feedback on patients’ experiences with the program, we tried to clarify further the relevance and the expected benefits for HCPs. All stakeholders and users were involved in the development of the program, and they all expect advantages of the program for patients with CKD. Although working with kidney disease is a challenge for many patients, these challenges concern a relatively small patient group since more than half of the patients with CKD are older than 65.

#### Users

In the CKD@Work study, it was already shown that HCPs endorse the importance of work-oriented clinical care. We aimed to further spread this belief in the department through increasing the awareness of other HCPs by informing and involving them from the start. The labor experts provided a short workshop for social workers, and we developed practical tools and the possibility to refer to the labor expert to equip HCPs with knowledge and skills and to increase self-efficacy for offering work-oriented care. Clarity in the hospital around who is responsible for which task was improved, so that HCPs gained confidence to carry out the program. Pilot data showed that both HCPs and patients were satisfied with WORK. Nevertheless, it remained unclear whether HCPs experienced personal benefit by applying the program and whether they experienced support from colleagues or supervisors. HCPs, in particular nephrologists, regularly indicated heavy workloads and therefore had to set priorities (sometimes have other goals and interests, e.g., conducting medical research or believing that work-oriented care is not per se part of being a doctor), which made adoption of the program more difficult.

#### End users (patients with CKD)

A lot of attention was paid to informing patients about the attention to work in healthcare, including by distributing brochures, discussion cards, and posters. The expectation was that this would provide patients with sufficient opportunity to become familiar with the WORK program and take advantage of it. However, only a small portion of all patients took the initiative to ask work-related questions to their doctor or another HCP. Patients who participated in WORK were very satisfied and really benefited from it.

#### Organization

Higher management in the hospital supported WORK, and the development was formally ratified. Most managers and HCPs at the department endorsed the need for work-oriented care. Delivering person-centered care is a part of the hospital’s vision statement; however, work-oriented care is not mentioned. Material resources and facilities, such as consulting rooms, were identified and made available for use in the program. Financial resources were available for the duration of the study; however, prolongation thereafter is unsure. Although we minimized the time required to provide work-oriented clinical care, some nephrologists still indicated that competing priorities in the hospital restricted the time available to apply the program. To encourage adoption, we identified early adapters and used them as engaged ambassadors who promoted the program within the department. Middle and higher management and HCPs were regularly informed about the program and information was made accessible to patients and HCPs. The COVID-19 pandemic measures forced HCPs and some members of the project group to temporarily be involved in care for COVID-19 patients. This turbulent phase did not help with the adoption and implementation of the program.

#### Socio-political context

The Dutch government has created a “participatory society” and emphasizes the need for all people to be self-reliant. A work-oriented clinical care program that supports patients to continue work participation despite illness fits well into this participatory approach. However, work-oriented clinical care and its financing has no formal basis yet. A financial barrier in the continuation of WORK is that funds that were previously available for healthcare innovation have been reduced and there is tremendous competition from costly technological innovation.

### Phase 6: Reflection and evaluation

#### Reflection on the process

A variety of hospital HCPs, patients from diverse backgrounds and external professionals who were already involved in occupational health were brought together to develop practical tools to help patients deal with work challenges. The chosen working method was labor intensive and time consuming. We admire the perseverance of all participants, the energy they had, and the knowledge they shared. Everybody was respectful to other parties and eager to learn from other perspectives, even though at some moments tension between different perspectives was felt. An important success factor of the project was the continuous team cooperation without anyone dropping out. All team members were dedicated throughout the project to produce results, reflect on preliminary findings, and continue to improve them. We feel that the organization of our project, with a dedicated Core team and an extended team with a diversity of participants, highly contributed to this continuous commitment. However, we also encountered some power dynamics during the project. Not all patients felt comfortable entering into discussion with high-educated professionals and expressing their ideas. In addition, tension was sometimes felt between external and internal participants at some moments. We dealt with this by additional communication between the Core team and the concerning participants.

As was mentioned earlier, the original development process (with live meetings and discussions) had to be adjusted due to the sudden emergence of COVID-19 restrictions. As the project continued, it was becoming clear that live meetings with the intended groups were not going to happen again. After two live meetings, all meetings were online using Microsoft Teams. In the early stages of the project not everyone was comfortable using Teams, however as time passed everyone became more comfortable. It is well possible that the enduring commitment to the project was facilitated by the online nature of the meetings, however we feel that live interaction in such an innovative project would have been more satisfactory for the participants.

### Evaluation

#### Feasibility and clinical utility

The feasibility and clinical utility of WORK was evaluated among patients with CKD, HCPs and hospital managers. Questions were focused on satisfaction, practicability, acceptability, accessibility, and comprehensibility.

#### Patients

Patients’ experiences with WORK were explored via a digital survey (Additional file 3). A total of 21 patients completed the digital survey (response: 33%), with a mean age of 46 years (range 23–61), and balanced gender representation. Sixty-six percent of the respondents did not have renal replacement therapy (medication, diet or pre-dialysis), 5% had hemodialysis, and 29% had undergone transplantation. Most patients were permanently employed (17/21) and had contact with an occupational health physician (16/21). A quarter of them did mentally demanding work, another quarter did physically demanding work, and the rest a combination of mentally and physically demanding work.

Patients assessed WORK with an average of 8.3 (with scores ranging from 5–10). All patients indicated that they find the attention for work in the hospital important (21/21) and the majority also reported that the doctor should discuss work with patients (16/21). The majority of patients (14/21) reported that the labor expert has given them more knowledge and motivation to continue working. About half indicated that they had started to do things differently with regard to work (9/19), had more control over their kidney disease (10/18), and had entered into a discussion with the employer (7/15). The majority of patients had also become aware of the obligatory steps that must be taken in re-integration during the first two years of illness (12/18). In all, the results of the evaluation of the clinical utility indicate that WORK may help patients to extend their capabilities to deal with work-related health conditions and allows them to become more self-directed. Most patients experienced WORK as a complete program (18/21). Some patients would have liked the information earlier in the disease trajectory or would have liked more or longer term support from the labor expert.

#### Health care professionals

Interviews were held with two hospital managers and nine HCPs, i.e. three nephrologists, three social workers, two nurses and the labor expert (Additional file 4). Average age of HCPs was 43.4 years (range 26–62 years) and on average they have 21.8 years of experience (range 5–39 years). They were asked to rate WORK on three components 1) the content, 2) the development and 3) the implementation.

The development of WORK was assessed with an average of 8 on a scale from 1–10. The way in which all stakeholders were involved in the development of WORK and the commitment of the project group were both assessed positively, although it was reported that the process was sometimes very demanding.

The content of WORK was also rated with an average score of 8. The involvement of the labor expert was positive, the flow diagram of the care pathway to target patients and tailor the support to their individual needs was pleasant and easy to use, and the three work questions were applicable. The other developed materials, such as the explanation about the Gatekeeper Improvement Act, were rarely used.

The implementation of WORK was assessed with an average score of 8.4. During a period of five months, 68 patients with CKD were referred to the labor expert. The participants indicated that the integration of the three work questions in the electronic patient files provided a reminder to pay attention to WORK. Some HCPs indicated that the referral to the labor expert could have been more user-friendly. They did not fully understand how it worked and needed explanation.

## Discussion

Many people with CKD have problems staying employed [12]. Patients and HCPs see the added value of work-oriented care, but so far this has received little attention in hospital care [16]. In collaboration with patients, doctors, nurses, social workers, and occupational and insurance physicians we developed and implemented work-oriented care in a hospital environment. We have focused on achieving two aims: 1) structurally embedding work-orientated care into the hospital, creating a culture in which the importance of work-oriented medical care is recognized by HCPs, and 2) strengthening the self-direction of patients with CKD for dealing with work challenges.

AIM was used for the systematic development, implementation, and evaluation of WORK. The process of developing WORK went well according to plan and timeframe, despite the difficulties arising from the COVID-19 pandemic. The final program was comprised of a care pathway including the targeting of patients (by the nephrologist or nurse), risk stratification, and tailored support. We anticipated needs from different stakeholder perspectives. The tools we developed may give HCPs more guidance to target patients who are struggling with work related issues and to refer these patients, if necessary, for appropriate support. The labor expert deployed for the project provided a point of contact at the outpatient clinic, which improved the accessibility of WORK. The practical tools may help patients to increase their self-management capabilities and to become well informed about the possibilities to work with CKD.

The implementation of WORK went well since most tools were used and appreciated both by HCPs and patients. Although all HCPs regarded the developed care path as rather easy and logical, an important barrier was the actual low readiness of some doctors to use the targeting tool. There is not always room for work-oriented care in nephrologists’ job perceptions. We argue that doctors have competing priorities during consultation and logically need to discuss the medical condition of the patients. Excess time to discuss work-related issues is often not available, which was also found by another study [31]. On the other hand, we noticed that a doctor’s personal motivation and ambition to provide work-oriented care was helpful to success. The will to provide work-oriented care was also present among the management, however the financial possibility to facilitate this was lacking. Financing work-oriented expertise (such as work provided by the labor expert) currently depends on the goodwill of the department or on temporary financing flows, such as project funds or innovation funds. Only a small portion of all patients took the initiative to ask work-related questions of their doctor. We argue that patients need time to realize that their hospital offers support on work challenges that might arise from their chronic illness. A culture change is not only needed at the HCPs and the hospital, but also among the patients. However, we stress that patients, beyond our scope, may use the practical tools and brochures to enhance their self-management capabilities and may feel no need to ask for additional support in the hospital.

A few limitations of this study should be mentioned. More than half of the people with CKD are 65 years or older and retired. As a result, the subject of "work" was perhaps less self-evident among the HCPs at the Nephrology Department. However, we expect that the results of this study can also be used in other diagnosis groups or other hospitals with a different context. A first exploration among the Departments of Endocrinology and Oncology shows that the need for work-oriented care might be even stronger there. Further, in this study the clinical utility of WORK was found positive, however, we have not yet been able to fully determine whether WORK leads to more self-direction and job retention. We must emphasize that a project of one and a half years is not long enough to structurally embed work-oriented care into the hospital. Within the available time frame, only the first steps of implementation could be taken. Embedding and continuation of this service of work-oriented care is a cultural change that takes more effort and time. We noticed that continuation of work-oriented clinical care needs a broader perspective and discussion about the scope of healthcare within the hospital, both on the department level and on the Board level. Management support may encourage such non-medical initiatives on the work floor. On a national level, we feel it is important that initiatives on work-oriented services in hospitals are combined and that a joint effort is undertaken to find out what is functioning best and what potential benefits it brings for patients and society. We feel this gap should be filled in the upcoming years as more people suffer from chronic illness and the labor market becomes tighter due to an aging population.

### Implications for practice

With the present program, work-oriented clinical care was implemented at the Department of Nephrology in the UMCG. Two other departments within the hospital (Endocrinology and Oncology) have already started using the program and supporting their patients with work-related challenges. This is a promising development for continuation, and it can help to make work-oriented care in the hospital a permanent part of healthcare. The knowledge acquired from this work-oriented program, including the tools that were developed, can also be easily converted and used by nephrology departments in other hospitals and for other groups of chronically ill patients.

The main challenges for implementation and continuation are that attention to work must fit into existing care structures, fit the needs of users, fit the vision of the organization, and become embedded in organizational policy. The awareness of HCPs in the hospital regarding work challenges may improve the early identification of patients at risk for prolonged time away from work and may allow for early supportive intervention over the entire care process of patients with CKD [12]. To achieve continuity, advocacy and lobbying are needed to establish the structural (financial) resources to maintain the program.

### Implications for research

Further research is needed to gain more insight into how patients can be empowered in returning to work from the hospital. To guide further development and implementation of the program, it will be necessary to explore how and under what circumstances outcomes are achieved. Therefore, the effectiveness of work-oriented clinical care could be examined using realistic evaluation [34]. Realistic evaluation is a theory-driven evaluation method that is increasingly used for studying the implementation of complex interventions within health systems. Realistic evaluation can be employed to explore how the program, with its specified performance- and change objectives, affects the outcomes.

## Conclusions

We developed and implemented WORK at the Department of Nephrology in a large hospital. Feasibility and clinical utility of WORK were rated positively. WORK offers HCPs in the hospital the necessary support and tools to empower patients with CKD to cope with work challenges. HCPs can discuss work in an early stage of care, support patients in anticipating potential work challenges, and bridge the gap to more specialized help if needed. So far, the implementation of the program has been successful, though structural implementation may be challenging.

## Supplementary Information

**Additional file 1.**

**Additional file 2.**

**Additional file 3.**

**Additional file 4.**

## Data Availability

The datasets generated and/or analyzed during the current study are not publicly available due to identifying information but are available from the corresponding author upon reasonable request.

## Abbreviations

AIM: Adapted intervention mapping
HCP: Health care professional
CKD: Chronic kidney disease
WORK: Work-oriented clinical care for kidney patients
UMCG: University Medical Centre Groningen
UWV: Institute for Employee Insurance
WIA: Employee benefits scheme
