# Supplementary material for: Development and implementation of work-oriented clinical care to empower patients with kidney disease: an adapted intervention mapping approach
Source: BMC Health Serv Res. 2023 Apr 1;23:329. doi: 10.1186/s12913-023-09307-9 (PMC10066946; doi:10.1186/s12913-023-09307-9)
Supplement: Supplementary file 1 — Additional file 1. [file 12913_2023_9307_MOESM1_ESM.docx]

**Additional file 1:** Core Team (centre), Taskforce and Advisory Board

**TASK FORCE**

[
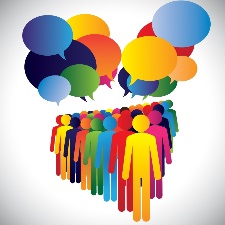

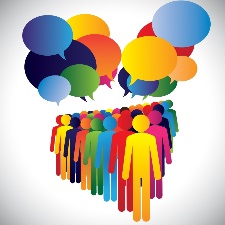
](https://www.google.nl/url?sa=i&rct=j&q=&esrc=s&source=images&cd=&cad=rja&uact=8&ved=2ahUKEwjxsd6KrKLgAhWEZ1AKHdj7CMsQjRx6BAgBEAU&url=https://www.effectmeting.nl/met-onderzoek-succesvolle-marketing-en-wervingsstrategieen-ontwikkelen/&psig=AOvVaw2DtYWhRRHmO-2vdbLr4DLy&ust=1549378830430117)

**ADVISORY BOARD**

Representatives of patients with CKD (Dutch Association for Kidney Patients); representatives of the Dutch Association of Occupational Medicine (NVAB), the Dutch Association for Insurance Medicine (NVVG), and the Dutch Association of labor experts (NVvA); representatives of The Confederation of Netherlands Industry and Employers (VNO-NCW), and researchers (National Network Chronically Ill and Work); Experts from hospitals, other initiatives of work-oriented care, and the Fit for Work platform.

[
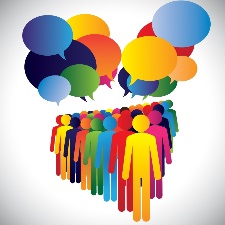

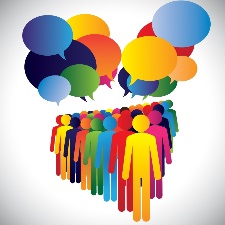
](https://www.google.nl/url?sa=i&rct=j&q=&esrc=s&source=images&cd=&cad=rja&uact=8&ved=2ahUKEwjxsd6KrKLgAhWEZ1AKHdj7CMsQjRx6BAgBEAU&url=https://www.effectmeting.nl/met-onderzoek-succesvolle-marketing-en-wervingsstrategieen-ontwikkelen/&psig=AOvVaw2DtYWhRRHmO-2vdbLr4DLy&ust=1549378830430117)
