# Supplementary material for: Development and implementation of work-oriented clinical care to empower patients with kidney disease: an adapted intervention mapping approach
Source: BMC Health Serv Res. 2023 Apr 1;23:329. doi: 10.1186/s12913-023-09307-9 (PMC10066946; doi:10.1186/s12913-023-09307-9)
Supplement: Supplementary file 2 — Additional file 2. [file 12913_2023_9307_MOESM2_ESM.docx]

**Additional file 2**: Matrix of change objectives for health care professionals and managers of the hospital, and for patients with CKD

| **Performance objectives hospital** | **Change objectives management and health care professionals** | | | | | |
| --- | --- | --- | --- | --- | --- | --- |
|  | **Knowledge** | **Skills** | **Attitude** | **Self-efficacy** | **Social influences** | **Facilities** |
| ***Health care professionals*** |  |  |  |  |  |  |
| *HCPs are aware of the value of work and the challenges patients with CKD face* | HCPs have knowledge and are aware of the value of work for patients, the difficulties patients face | - | - | - | - | - |
| *HCPs have knowledge and possibilities to deliver work-oriented care or to refer patients* | HCPs have knowledge of the way in which work-oriented care has been organized within the department | HCPs feel competent and have the possibilities and tools to offer tailored work-oriented clinical care and to recognize risk factors for (future) problems at work | HCPs accept and are positive about the work-oriented care as defined | HCPs feel confident with and believe in their ability to pay attention to work | HCPs and management support colleagues to pay attention to WORK | HCPs have time to pay attention to work |
| *HCPs know and agree with the tasks and roles they have in providing work-oriented clinical care* | Nephrologists and nurses know that they can refer patients easily to the social worker or the labor expert based on three ‘work-questions’ | HCPs are able to recognize their task in work-oriented clinical care | HCPs in the hospital are motivated to take their role in work-oriented care | HCPs feel confident about their expertise to take their role in work-oriented care | HCPs in the hospital are discussing work-related care with colleagues and putting work-oriented clinical care on the agenda | - |
| ***Organizational level*** |  |  |  |  |  |  |
| *Managers create a culture to deliver work-oriented care* | Managers are increasingly aware of the value of work for patients their role in supporting HCPs in paying attention to work | - | Managers are motivated to put work-oriented care on the agenda and consider this as part of their job | - | Managers’ attention to work a topic for discussion among HCPs as well as among members of the higher management and the board of directors | - Managers offer HCPs time, place to work and tools to pay attention to work - Referral to a labor expert in the hospital is facilitated - Attention for work is incorporated in existing working methods |
| **Performance objectives patients with CKD** | **Change objectives patients with CKD** | | | | | |
|  | **Knowledge** | **Skills** | **Attitude** | **Self-efficacy** |  |  |
| *Increasing awareness and use of work-oriented-care* | Patients with CKD know that they can go to the HCP in the hospital with questions about work | Patients with CKD are able to use work-oriented support in the hospital | Patients with CKD are positive about the work-oriented care that is offered | - |  |  |
| *Empowering patients to handle work challenges* | Patients with CKD are aware of the circumstances that make work easier or more difficult for them, for example the laws and regulations that are relevant to them.  Patients with CKD know own responsibilities and what to expect from stakeholders in the re-integration process  Gaining insight in own health status and addressing interference of treatment and work  Patients with CKD know who to turn to for support | - Patients with CKD are able to cope with work-related challenges, such as finding a balance between load and load capacity, the possibility of work adjustments - Patients with CKD are able to ask questions to seek help to handle work related challenges, for example by asking HCP, the labor expert or the occupational health physician - Patients with CKD are able to decide whether or not to disclose their disease at work - Patients with CKD are able to prepare a consult with the OHP | Patients with CKD are aware of own role and responsibility in handling work challenges | Patients with CKD feel confident and capable to handle work related-challenges |  |  |

HCPs=health care professionals; CKD=chronic kidney disease.
