# Supplementary material for: Development and implementation of work-oriented clinical care to empower patients with kidney disease: an adapted intervention mapping approach
Source: BMC Health Serv Res. 2023 Apr 1;23:329. doi: 10.1186/s12913-023-09307-9 (PMC10066946; doi:10.1186/s12913-023-09307-9)
Supplement: Supplementary file 3 — Additional file 3. [file 12913_2023_9307_MOESM3_ESM.docx]

**Additional file 3: Survey among patients with chronic kidney disease (CKD)**

This is a selection of the most relevant topics that have been asked in the survey.

*Introduction*

1. Do you have paid work?

o Yes, namely

o No, I am looking for work (go to question 5)

o Other, namely …………

2. How many hours per week do you work (according to contract?) ………..

3. What kind of work do you mainly do?

o Mentally demanding work

o Physically demanding work

o Both

*Experiences with work-oriented clinical care for patients with CKD (WORK)*

1. How satisfied are you with the work-oriented clinical care that you received?

Score 1-10 (1= very dissatisfied, 10 = very satisfied)

Explanation …………………

2. Did the care and/or information provided meet your needs?

Yes because …….

No, because …….

3. Are there things you missed?

Yes because …….

No, because …….

*Response categories: Strongly disagree – Disagree – Neutral – Agree - Strongly agree*

*Acceptability*

1. I think it is important that the hospital pays attention to work

2. I think the doctor in the hospital should talk to his/her patients about work

3. I think that the health care providers in the hospital are sufficiently knowledgeable to discuss questions about work

*Clinical utility*

*Response categories: Not at all true - Not true – Neutral - True - Very true*

Due to the focus on work from the hospital,

1. … I started doing things differently concerning work

2. … I feel I have a better grip on working with my illness

3. … I talked to my employer

4. … I know more about the steps that have to be taken when you call in sick

*Closing*

1. What is your age?

2. What is your gender?

3. At which department are you being treated?

o Nephrology UMCG

o Dialysis Center Groningen (DCG)

o Other, namely ………
