# Supplementary material for: Development and implementation of work-oriented clinical care to empower patients with kidney disease: an adapted intervention mapping approach
Source: BMC Health Serv Res. 2023 Apr 1;23:329. doi: 10.1186/s12913-023-09307-9 (PMC10066946; doi:10.1186/s12913-023-09307-9)
Supplement: Supplementary file 4 — Additional file 4. [file 12913_2023_9307_MOESM4_ESM.docx]

**Additional file 4: Interview schedule HCPs and managers**

*Introduction*

1. What is your age?

2. What is your position at work?

3. How many years of experience do you have in nephrology healthcare?

*Opening questions*

1. Do you pay attention to work in your contact with patients? Why or why not?

2. How do you pay attention to work?

3. Did you do this in a different way before the development and implementation of WORK in the department and if so, how did you do it then?

*Key questions*

*Acceptability*

1. Do you consider work-oriented care/attention to work as part of your duties as a doctor/nurse/social worker?

2. Do you feel that management considers attention to work to be important? If so, what does this show? If not, what does this have to do with?

3. Do you feel that your colleagues find attention to work important? If so, what does this show? If not, what does this have to do with?

4. How central do you think work should be in healthcare practice?

*Practicability*

1. Which elements of WORK did you use and how often? (were the three work questions asked and/or have patients been referred to a social worker or labor expert?)

2. What are your experiences with the labor expert and does the use of a labor expert in the hospital have added value for the care provided to the patient?

*Accessibility*

1. Did you experience working with WORK easy or difficult? And what made working with WORK easy or difficult? (e.g. were resources easy to find, were questions easy to ask, etc.)

2. If YES to question 1 of practicability: And if so, was the referral to social worker/labor expert and the tools developed easy to use and/or accessible?

If NO to question 1 of practicability: And if not, what were the obstacles you experienced with these elements of WORK?

*Applicability*

1. Does the focus on work have added value for your work as a healthcare provider?

2. Does the focus on work have added value for patients?

3. Do you plan to continue the focus on work in your patient care? Why/why not? What makes it easier or more difficult for you to do or not to do this?

4. Do you have any important tips for how the implementation of a project like this can be shaped in the future?

*Closing*

1. These were the questions I wanted to ask. Is there anything else you'd like to add?

2. Do you have any questions for me?

3. In any case, thank you very much for your time and effort in participating in this research. Are you open to me contacting you later for a few additional questions?

*Additional questions for managers*

1. As a manager, do you think that attention to work belongs in the hospital?

2. How important do you think the attention to work is, and why?

3. How do you think HCPs feel about work-oriented care in the hospital?

4. What do you think is necessary to ensure that WORK can be integrated into the existing system of care provision in the hospital?
